# Regulating bulb dormancy release and flowering in lily through chemical modulation of intercellular communication

**DOI:** 10.1186/s13007-023-01113-y

**Published:** 2023-11-28

**Authors:** Yajie Zhao, Wenqiang Pan, Yin Xin, Jingxiang Wu, Rong Li, Jinxin Shi, Shuo Long, Lianwei Qu, Yingdong Yang, Mingfang Yi, Jian Wu

**Affiliations:** 1 Beijing Key Laboratory of Development and Quality Control of Ornamental Crops, Department of Ornamental Horticulture, China Agricultural University, No. 2 Yuanmingyuan West Road, Haidian, Beijing, 100193 China; 2 Institute of Floriculture, Liaoning Academy of Agricultural Sciences, Shenyang, 110161 China

**Keywords:** Lily, Bulb dormancy release, Flowering, Symplastic transport, BDM, NEM, DDG

## Abstract

Lily is a bulbous plant with an endogenous dormancy trait. Fine-tuning bulb dormancy release is still a challenge in the development of bulb storage technology. In this study, we identified three regulators of symplastic transport, 2,3-Butanedione oxime (BDM), N-Ethyl maleimide (NEM), and 2-Deoxy-D-glucose (DDG), that also regulate bulb dormancy release. We demonstrated that BDM and DDG inhibited callose synthesis between cells and promoted symplastic transport and soluble sugars in the shoot apical meristem (SAM), eventually accelerating bulb dormancy release and flowering in lilies. Conversely, NEM had the opposite effect. These three regulators can be flexibly applied to either accelerate or delay lily bulb dormancy release.

**Supplementary Information:**

The online version contains supplementary material available at 10.1186/s13007-023-01113-y.

## Introduction

Lily bulb dormancy is classed into bud dormancy, and bulb dormancy release should be carried out under low temperatures. Excessive cold storage time can lead to necrosis of the central bud while insufficient storage time can result in several severe issues, including uneven seedling emergence, stunted growth, blind flowers, rosette plants, delayed flowering, reduced flower buds, and flower abortion. Therefore, fine-tuning dormancy release has become a crucial challenge in the postharvest of lily bulbs and has emerged as a prominent topic in lily research. Bud dormancy is regulated by various factors, including environmental factors, endogenous hormones, metabolic substances, and epigenetic modifications [1–5].

Intercellular communication via plasmodesmata (PD) plays a crucial role during bud dormancy released in woody plants and bulbous plants [6, 7]. PD serves as a communication channel between the cytoplasm of adjacent cells, with an opening running through the cell walls. The cells connected by PD, known as symplasts, facilitate intercellular communication and molecular exchange, distinguishing them from the parts that are not connected (apoplasts). This communication mechanism through intercellular PD is crucial for plant growth and development [8]. It allows for the movement of various substances, such as small molecules like sugars, ions, and essential nutrients, as well as proteins, different types of RNA complexes, and other macromolecules, bridging adjacent cells across cell walls. PD serves as a multifunctional channel with varying numbers and structures, and its permeability is continuously adjusted in response to various internal and external factors [9]. In potatoes, chilling could induce the sugar accumulation and further reduced PD closure [10]. The blockage of PD result in the tuber dormancy by reducing tuberigen proteins and sucrose transport [11].

Actin and myosin are fundamental elements of the cytoskeleton in plant cells that are located in cytoplasmic channels [12, 13]. During the early stages of cell growth and differentiation, actin and myosin play a role in regulating PD and controlling its permeability. Myosin ATPase inhibitor 2,3-Butanedione monoxime (BDM) induces the separation of myosin and actin at the cell membrane and endoplasmic reticulum to open the PD. As for myosin inhibitor N-Ethylmaleimide (NEM), in some plant tissue, it was found to inhibit actin–myosin-mediated organelle movement and cytoplasmic streaming, to promote the firm attachment of myosin and actin and to close the PD [13–15].

Callose is a β-1,3-glucan polysaccharide involved in plant development and stress responses, such as the dynamics of PD and sieve pores, pollen development, vascular differentiation, the cell plate formation, and responses to biotic and abiotic stresses [16, 17]. The movement of a large number of molecules between cells through PD is regulated by callose-dependent and non-callose-dependent mechanisms [18–20]. Non-callose-dependent mechanisms involve changes in PD density and structure, such as the transition from a simple branching form to a complex branching form, the participation of actin, and PD permeability [18, 21]. In the callose-dependent pathway, the dynamic regulation of callose content in the neck region of PD influences the opening and closure of PD channels. High callose content leads to the closure of PD channels, while low callose content promotes PD channel opening [22]. During cell division, callose is deposited on the cell plate but subsequently degraded after cell division is complete [23]. 2-Deoxy-D-glucose (DDG) was used as a callose synthesis inhibitor that helps to alleviate callose deposition at PD by inhibiting callose synthesis [24]. The callose biosynthesis gene, *CALLOSE SYNTHASE* (*CALS*), is involved in plant development and stress response. During plant morphogenesis in Arabidopsis, overexpressing *CALS* leads to defects in root development due to closed PD, which limits the movement of transcription factor SHORT-ROOT and *microRNA165* between stele and the endodermis via the PD [25]. In *Populus*, increased *CALS1* expression results in closed PD which blocks intercellular communication and slows down plant growth during the dormancy-inducing stage [6]. In lilies, *LoCALS3* negatively regulates bulb dormancy release by increasing callose deposition at PD in SAMs [7].

In this study, we aimed to investigate the role of PD by applying chemical regulators using a new vacuum method. We treated the lily bulbs with different symplastic transport regulators, NEM, BDM, and DDG, when they are dormant. The results showed that the vacuum treatment was an efficient way to feed the bulb; BDM and DDG promoted bulb dormancy release and flowering, similar to the function of additional cold treatment; NEM had the opposite effect, inhibiting bulb dormancy release and plant growth.

## Result

### Controlling bulblets/bulbils growth: NEM suppression, BDM & DDG enhancement

**Fig. 1:**
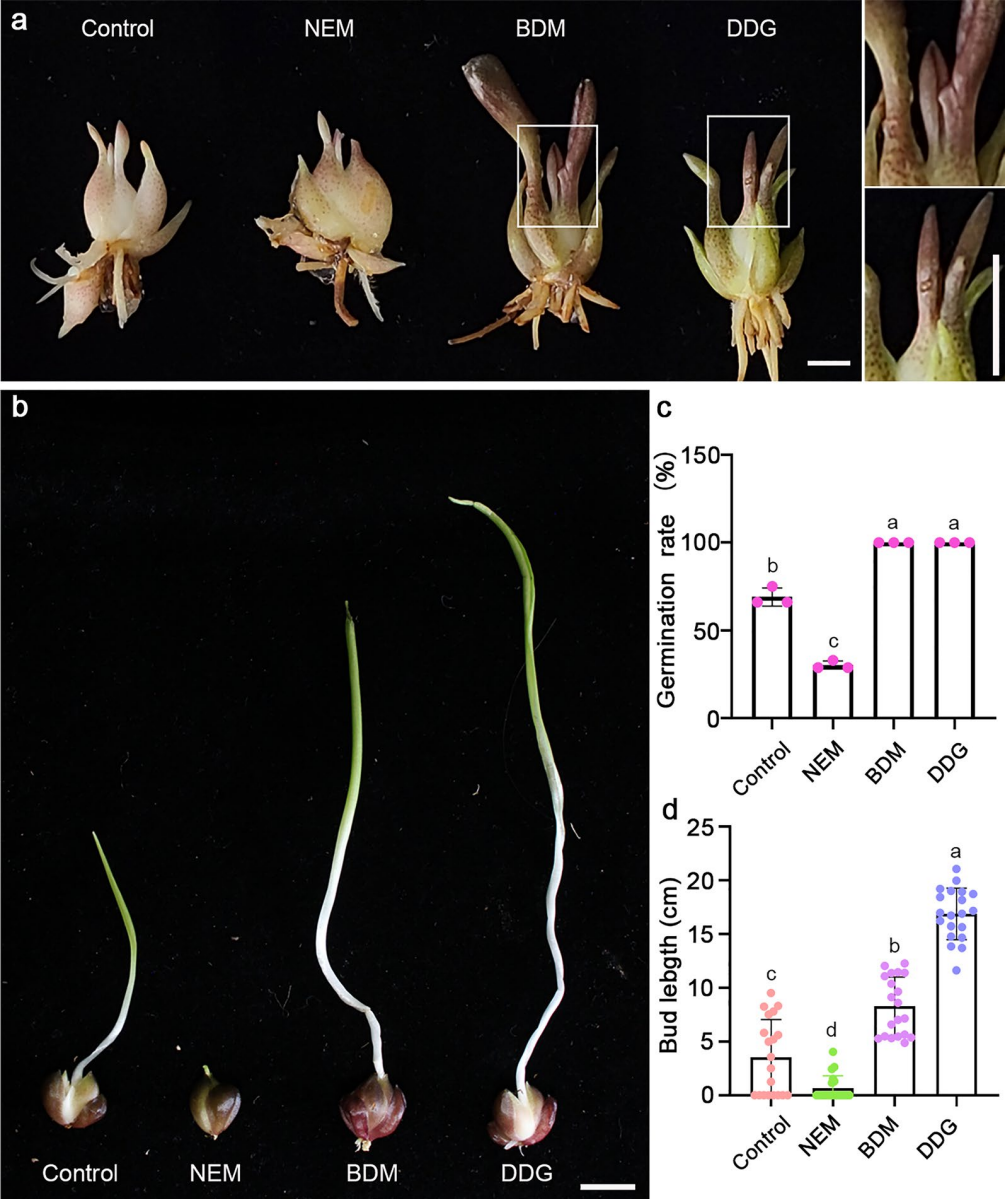
Effects of NEM, BDM, and DDG on the sprouting of Siberia bulblets and *L. lancifolium* bulbils. **a** Phenotypes of Siberia bulblets treated with NEM, BDM, and DDG for 6 weeks. Scale bars, 1 cm. The right panels are enlarged images showing the sprouted leaves of bulblets with BDM (upper) and DDG (lower) treatments. **b** Phenotypes of bulbils of *L. lancifolium* with NEM, BDM, and DDG treatments 20 weeks after planting on soils. Scale bars, 1 cm. **c** The germination rate of bulblets after 6 weeks of cultivation on MS medium with NEM, BDM, or DDG treatment. MS medium served as the control. A bulblet with over 1 cm of leaf was counted as a sprouted bulblet. The data represent mean ± s.d. of 3 biological replicates (n = 10 bulblets per sample). **d** Bud length of bulbils 20 weeks after planting. Bulbils were treated with NEM, BDM, or DDG before potting on soil. The data represent mean ± s.d. of 20 independent bulbils. Different letters indicate significant differences (*p* < 0.05) by ANOVA Turkey’s HSD tests for pairwise comparisons

To investigate the roles of NEM, BDM, and DDG on the growth of buds, both dormant bulblets of lily cultivar Siberia and bulbils of *Lilium* *lancifolium* were treated with these regulators (Additional file 1: Fig. S1). The results were consistent with a previous study [7], bulblets treated with DDG and BDM were able to sprout earlier, while NEM treatment delayed the germination (Fig. 1a). For bulbils, after 20 weeks, all bulbils with BDM and DDG treatments sprouted with developed leaves, while the NEM lines barely germinated (Fig. 1b–d). These results indicate that BDM and DDG can promote the germination of different propagation organs of the lily while NEM has opposite effects.

### NEM, BDM, and DDG regulate callose deposition in the PD

**Fig. 2:**
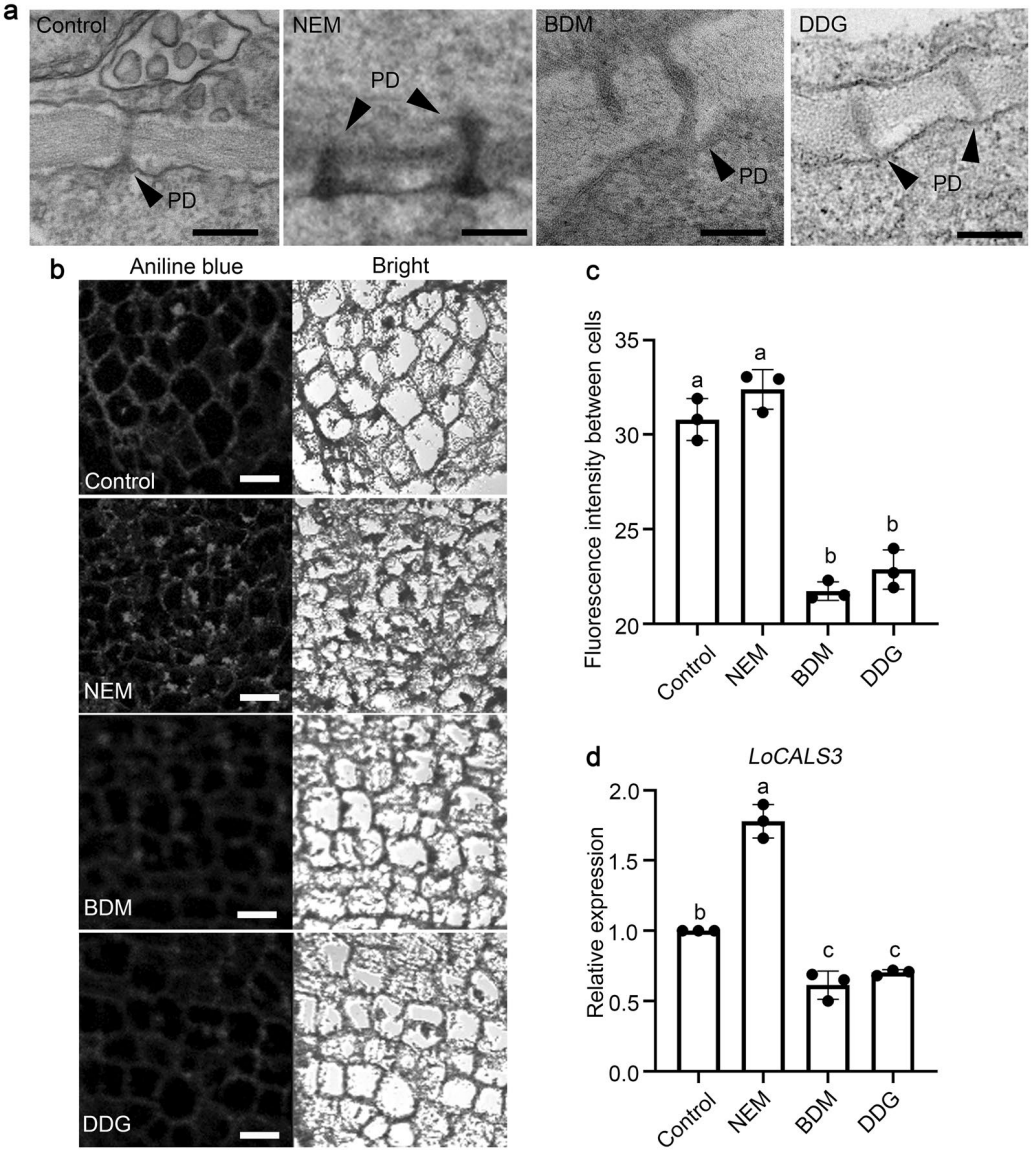
Effects of NEM, BDM, and DDG on callose deposition of SAMs in Siberia bulblets. **a** TEM microscopy micrographs of SAMs of lily bulblets 6 weeks after treatment with NEM, BDM, and DDG. SAMs of Control and NEM treatment exhibit electron-dense dormancy sphincters associated with PD, while BDM or DDG treatment lacks this structure. Similar results were observed in three independent samples. PD, Plasmodesmata. Scale bars, 0.2 μm; **b** Callose deposition between cells in SAMs of lily bulblets 6 weeks after treatment with NEM, BDM, and DDG. SAMs were subjected to aniline blue staining. Scale bars, 40 μm. Consistent results were obtained in 3 independent bulblets; **c** Callose levels were decreased in SAMs of BDM and DDG-treated bulblets and maintained high levels in NEM-treatment bulblets. Aniline blue staining and ImageJ software were used for quantification. Data are presented as mean ± s.d. of three biological replicates (n = 3 slices per replicate). Different letters indicate significant differences (*p * < 0.05) by ANOVA Turkey’s HSD tests for pairwise comparisons. **d** The expression of *LoCALS3* in SAMs of bulblets 6 weeks after treatment with NEM, BDM, and DDG. Data are presented as mean ± s.d. of three biological replicates (n = 3 SAMs per sample). Different letters indicate significant differences (*p* < 0.05) by ANOVA Turkey’s HSD tests for pairwise comparisons

Given that NEM, BDM, and DDG regulate callose deposition at PD in *Tradescantia*, *Pisum sativum*, and *Lilium spp*. [7, 13, 26], we conducted further investigations into the effect of these compounds on callose deposition and PD aperture in SAMs of bulblets using transmission electron microscopy (TEM) and aniline blue staining. Our findings consistently showed that more callose was deposited around PD channels in NEM-treated SAMs while PD remained open in BDM/DDG-treated SAMs (Fig. 2a). Additionally, callose content between cells in BDM/DDG-treated SAMs decreased by approximately 30% compared to the control (Fig. 2b, c). Interestingly, 1 mM NEM treatment maintained callose content in SAMs similar to the control, suggesting that bulbs in these two groups were still dormant and the callose was not degraded yet (Fig. 2b, c). Furthermore, we investigated the expression of an essential callose synthesis gene, *LoCALS3* [7], in SAMs of these treated bulblets. Compared to the control, *LoCALS3* expression was downregulated in BDM/DDG-treated bulblets while upregulated in NEM-treated bulblets (Fig. 2d). These results are in line with the sprouting phenotypes observed in Fig. 1a.

### NEM, BDM, and DDG affect the import of soluble sugar in SAM of bulblets

**Fig. 3:**
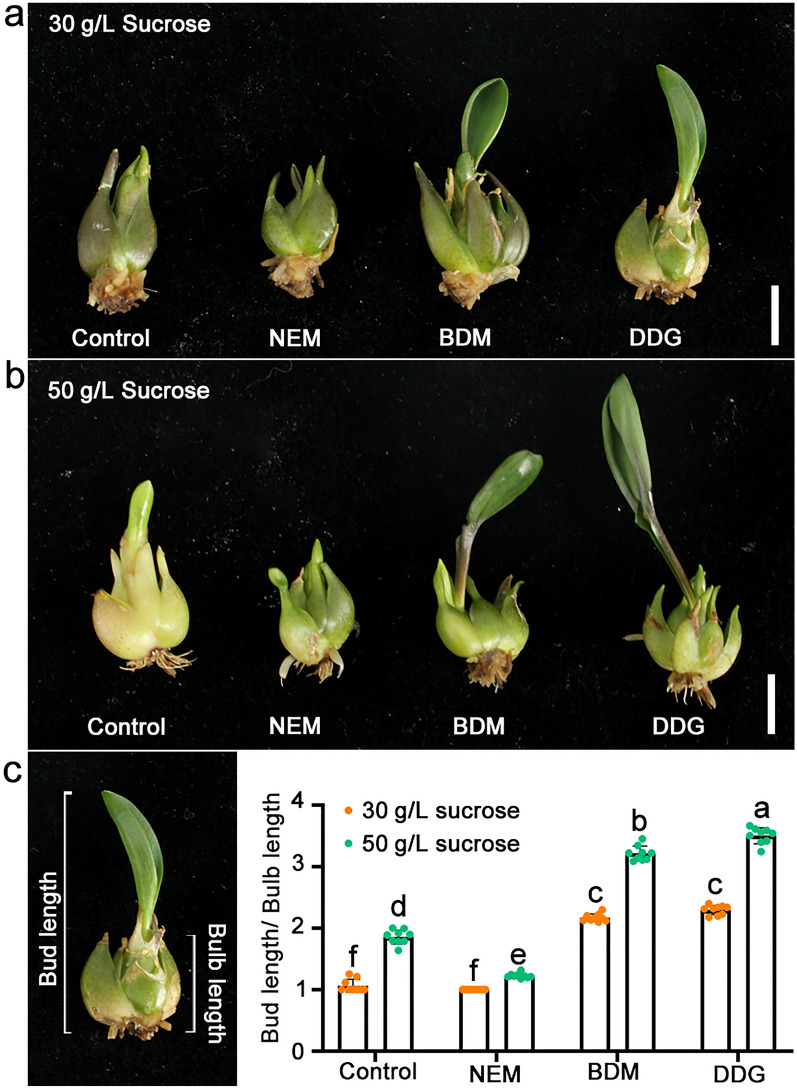
Effects of sucrose in combination with NEM, BDM, and DDG on the sprouting of Siberia bulblets. **a**, **b** Phenotypes of dormant Siberia bulblets grown on MS media containing 1mM NEM, 1mM BDM, or 1mM DDG in combination with either 30 g/L sucrose (**a**) or 50 g/L sucrose (**b**) for a duration of 5 weeks. The bulblets were grown in a growth chamber under 16/8 h light/darkness 22 °C/18°C. Scale bars, 1 cm. **c.** The bud-to-bulb length ratio of bulblets after 5 weeks of treatments. The data represent mean ± s.d. of nine independent bulblets. Different letters indicate significant differences (*p* < 0.05) by ANOVA Turkey’s HSD tests for pairwise comparisons

As sugars are important energy sources for dormancy release [27], we initially observed the phenotypes of dormant lily bulblets following treatment with various concentrations of sucrose combined with different regulators. Compared to the control (30 g/L sucrose), a higher sucrose concentration (50 g/L) accelerated bulb dormancy release (Fig. 3). When combined with BDM or DDG, the higher sucrose concentration further enhanced bulblet sprouting (Fig. 3). Sucrose could also partially alleviate the inhibition effect of NEM on sprouting (Fig. 3). These results show that sucrose positively regulates bulb dormancy release and are consistent with the aforementioned result that BDM and DDG promote bulb sprouting, while NEM exerts inhibitory effects (Figs. 1 and 3).

**Fig. 4:**
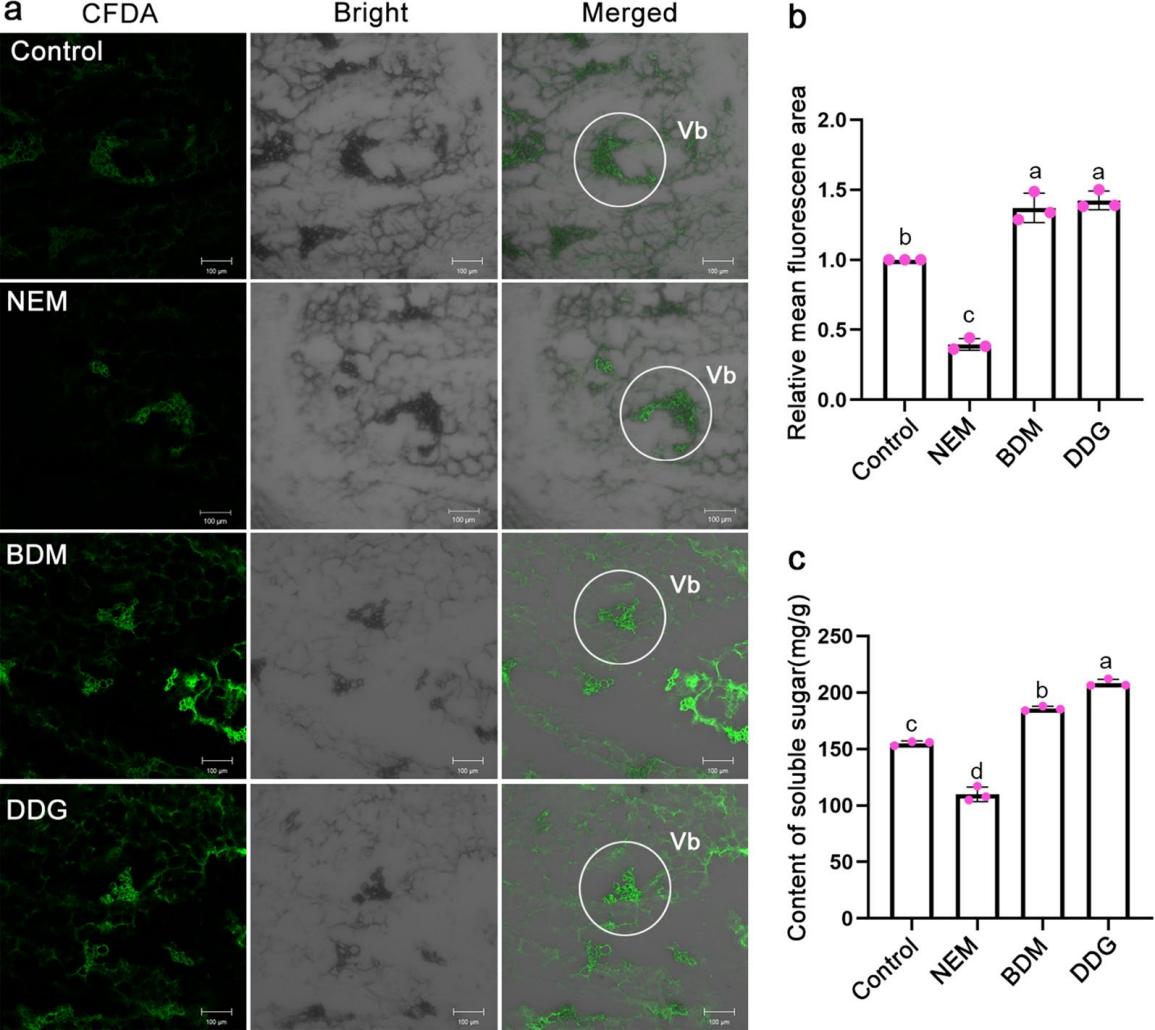
Effects of NEM, BDM, and DDG on sugars transport in SAM of Siberia bulblets. **a**, **b** Simulation of symplastic transport by CFDA in central buds of NEM, BDM, and DDG-treated bulblets (**a**). Compared to the control (ddH_2_O), CFDA was less expanded to the surrounding parenchyma cells under NEM treatment, while BDM and DDG promoted CFDA expansion (**b**). The green fluorescence of CFDA and ImageJ software were used for quantification. Scale bars, 100 μm, Vb: vascular bundles. **c** Soluble sugar was measured in SAMs of bulblets 6 weeks after the treatments with NEM, BDM, and DDG. The data in panels **b** and **c** represent mean ± s.d. of 3 biological replicates (n = 3 slices/SAMs per sample). Different letters indicate significant differences (*p* < 0.05) by ANOVA Turkey’s HSD tests for pairwise comparisons

Since sugars can be delivered from source to sink organs via symplastic transport [28], we mimicked the symplastic transport of sugars by using carboxyfluorescein diacetate (CFDA) and also analyzed the soluble sugars in BDM/DDG/NEM-treated SAMs. The results showed that compared to the control, BDM and DDG could promote symplastic transport with larger fluorescence areas, while NEM had the opposite effect (Fig. 4a, b**)**. Given that sugars can also be transported via apoplastic transport, we tracked the apoplastic transport of sugars in those treated SAMs by using esculin staining. However, no significant difference in fluorescence in all treatments, suggesting BDM, DDG, and NEM may not affect the apoplastic transport of sugars (Additional file 1: Fig. S2). Soluble sugars in BDM/DDG-treated SAMs were significantly higher, while NEM-treated SAMs had lower levels (Fig. 4c; Additional file 1: Fig. S3).

In addition, *FLOWER LOCUS T* (*FT*) message (m)RNA can be carried to SAMs via symplastic transport in lily [7], we examined *LoFT1* expression in the SAM of bulblets by RT-qPCR. The accumulation of *LoFT1* mRNA was higher in BDM/DDG-treated SAMs while lower in NEM-treated SAMs (Additional file 1: Fig. S4).

In all, these results (Figs. 2 and 4) suggest that BDM and DDG stimulate sugar import in SAMs via enhanced symplastic transport by reducing callose deposition at PD, while NEM exhibits the opposite effect.

### NEM, BDM, and DDG affect the lily bulb dormancy released

**Fig. 5:**
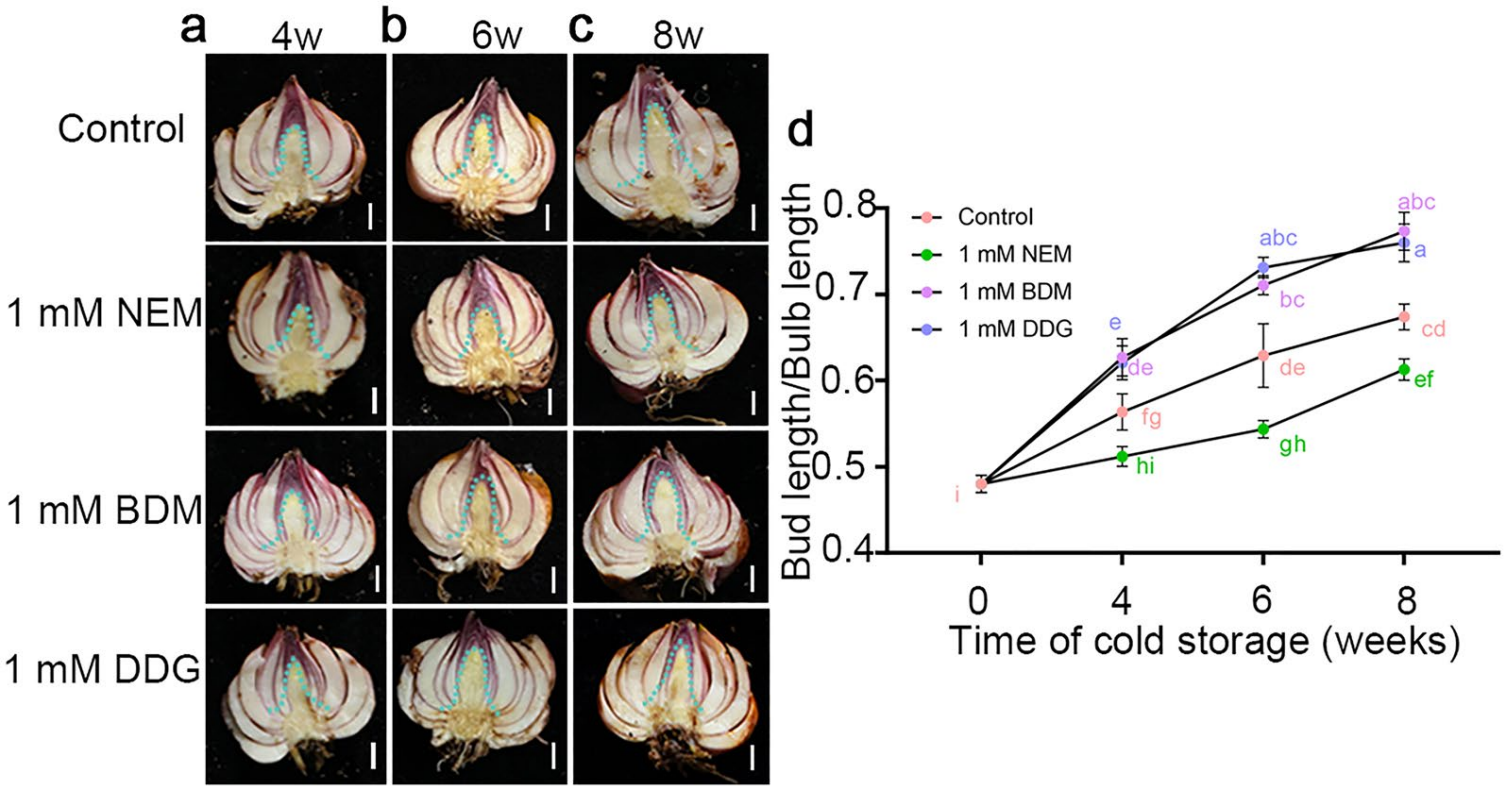
Effects of NEM, BDM, and DDG on bulb dormancy release in Siberia. **a–c** Bud elongation in dormant Siberia 4 weeks (**a**), 6 weeks(**b**), and 8 weeks (**c**) of the low-temperature storage (4 °C) after being treated with NEM, BDM, and DDG. Water treatment was used as the control. The blue dotted line outlined the position of the central bud. Scale bars, 1 cm; **d** The central bud-to-bulb length ratio. Data represent mean ± s.d. of three biological replicates (n = 8 bulbs per sample). Different letters indicate significant differences (*p* < 0.05) by ANOVA Turkey’s HSD tests for pairwise comparisons

Siberia bulbs are exclusively used for cut-flower production and undergo 2–3 months of low-temperature storage to break dormancy before planting. Considering that commercial Siberia bulbs (ΦA > 5 cm) are significantly larger than bulblets (ΦA = ~ 1 cm), we aimed to determine if the treatment method used for bulblets could also be applied to the commercial bulbs. To address this, we used the same short-term bulbil treatment for Siberia dormant bulbs (ΦA = 6–7 cm) and analyzed the elongation of central buds following low-temperature storage for 4, 6, and 8 weeks (Fig. 5; Additional file 1: Fig. S5). Our results demonstrated that the treatment protocol was effective for the commercial bulbs, yielding similar effects. Specifically, 1 mM BDM and 1 mM DDG significantly promoted bud elongation, while 1 mM NEM repressed elongation (Figs. 1 and 5). These findings suggest that the regulatory effects of these compounds on lily bulb dormancy release are consistent and hold promise for practical applications in the cut-flower industry.

### NEM, BDM, and DDG affect bulb sprouting and plant growth

**Fig. 6:**
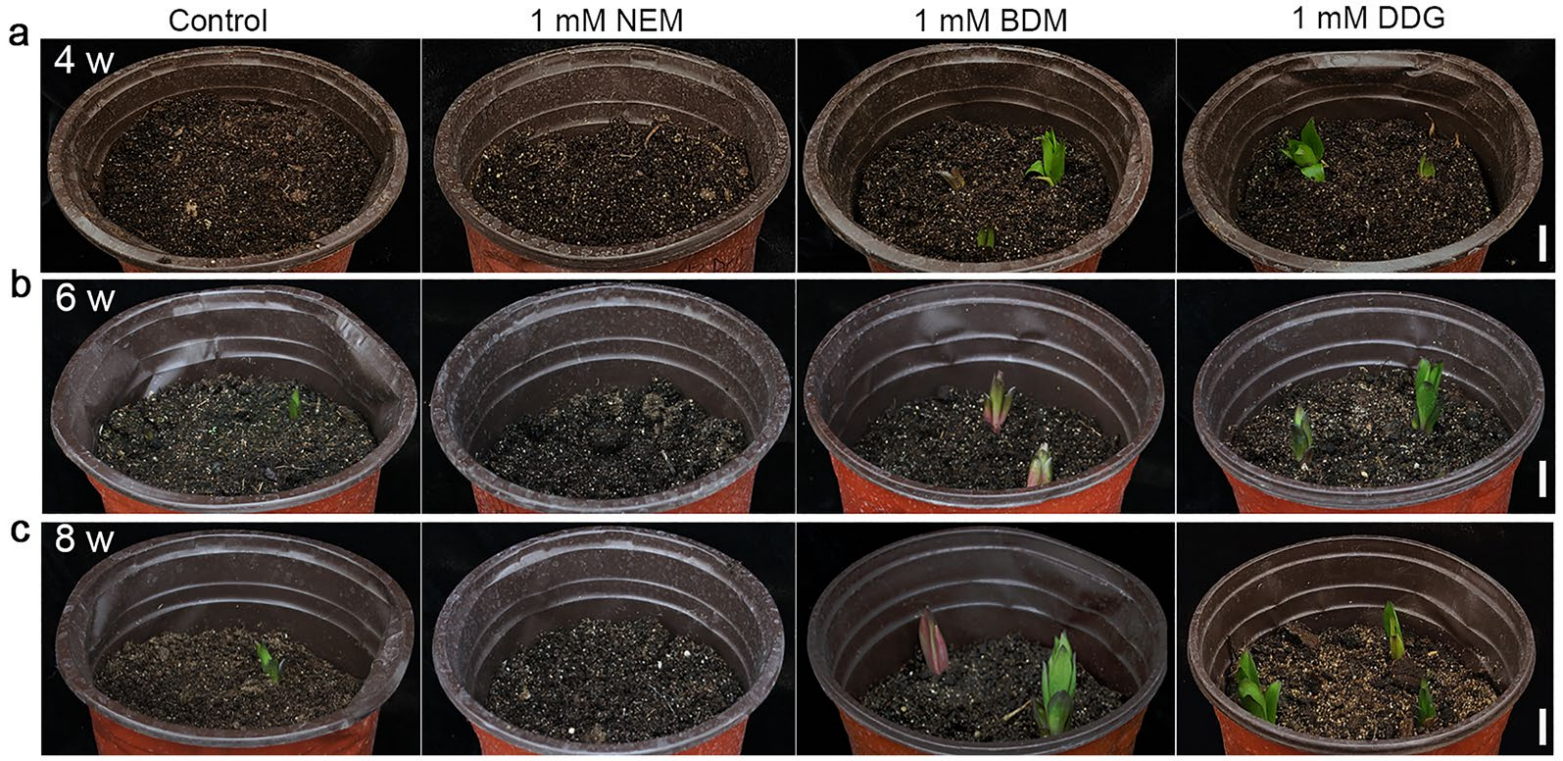
Effects of NEM, BDM, and DDG on sprouting of dormant Siberia bulbs. **a–c** Sprouting test was conducted for dormant Siberia bulbs after treatment with NEM, BDM, and DDG, followed by storage under the low temperature (4 °C) for 4 weeks (**a**), 6 weeks (**b**), and 8 weeks (**c**). The images were taken 8 weeks after planting for **a**, 5 weeks after planting for **b**, and 3 weeks after planting for **c**. Scale bars, 3 cm; Six bulbs were used in each sample. Consistent results were obtained in 3 biological repeats

**Fig. 7:**
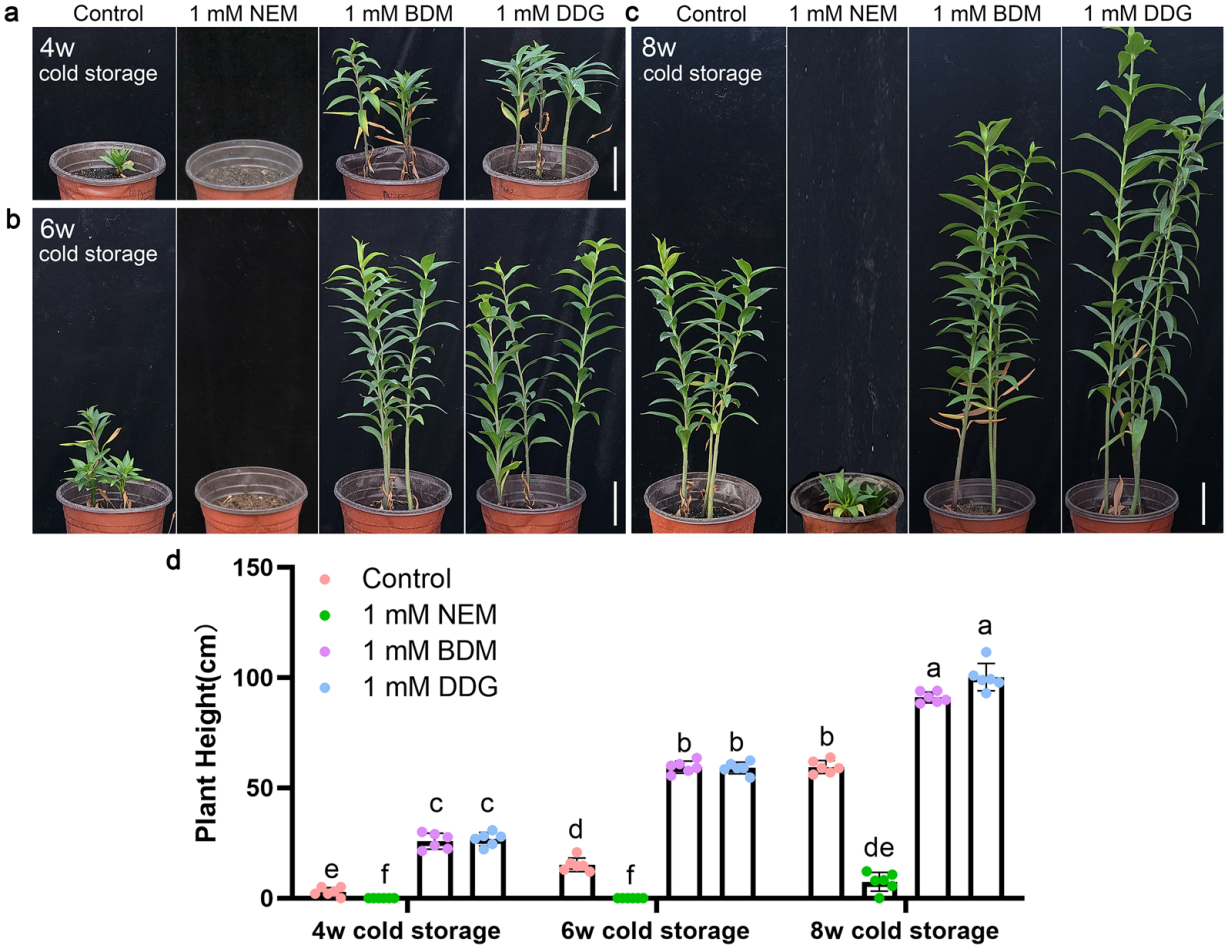
Effects of NEM, BDM, and DDG on plant growth. **a–c** Plant growth was analyzed for dormant Siberia bulbs after treatment with NEM, BDM, and DDG, followed by storage under the low temperature (4 °C) for 4 weeks (**a**), 6 weeks (**b**), and 8 weeks (**c**). All images were taken 16 weeks after planting. Scale bars, 5 cm; **d** Plant height was measured 16 weeks after planting. Data represent mean ± s.d. of six biological replicates (n = 6 bulbs per sample). Different letters indicate significant differences (*p* < 0.05) by ANOVA Turkey’s HSD tests for pairwise comparisons

Normally, Siberia bulbs require 8 weeks of cold storage (4 °C) to release bulb dormancy. Insufficient cold storage time resulted in stunted growth. To determine whether BDM and DDG can complement the stunted growth caused by insufficient cold storage time, we treated dormant bulbs with BDM, DDG, or NEM before storing them at 4 °C. (1) When bulbs had acquired half of the cold storage (4 weeks), the control bulbs barely sprouted and only a few developed into rosettes 16 weeks after planting. Meanwhile, BDM/DDG-treated bulbs sprouted and reached an appropriate height of 25 cm but the NEM-treated bulbs remained dormant (Figs. 6 and 7a, d). (2) When bulbs were stored for 6 weeks, the control bulbs were able to sprout but the plants remained dwarfed. In contrast, BDM/DDG-treated bulbs showed promoted growth and developed into tall plants, while the NEM-treated bulbs were still dormant (Figs. 6 and 7b, d). (3) When the control bulbs were released from dormancy after 8 weeks of cold storage, BDM/DDG-treated bulbs were still taller than the control, while the NEM-treated bulbs showed a rosette phenotype (Figs. 6 and 7c, d). Notably, based on the plant height, the effect of 1mM BMD/DDG was roughly equal to 2 weeks of cold storage, suggesting BDM and DDG play positive roles in recovering from the plant arrest caused by insufficient cold storage time.

### BDM and DDG can partially replace cold treatment for the flowering trait

**Fig. 8:**
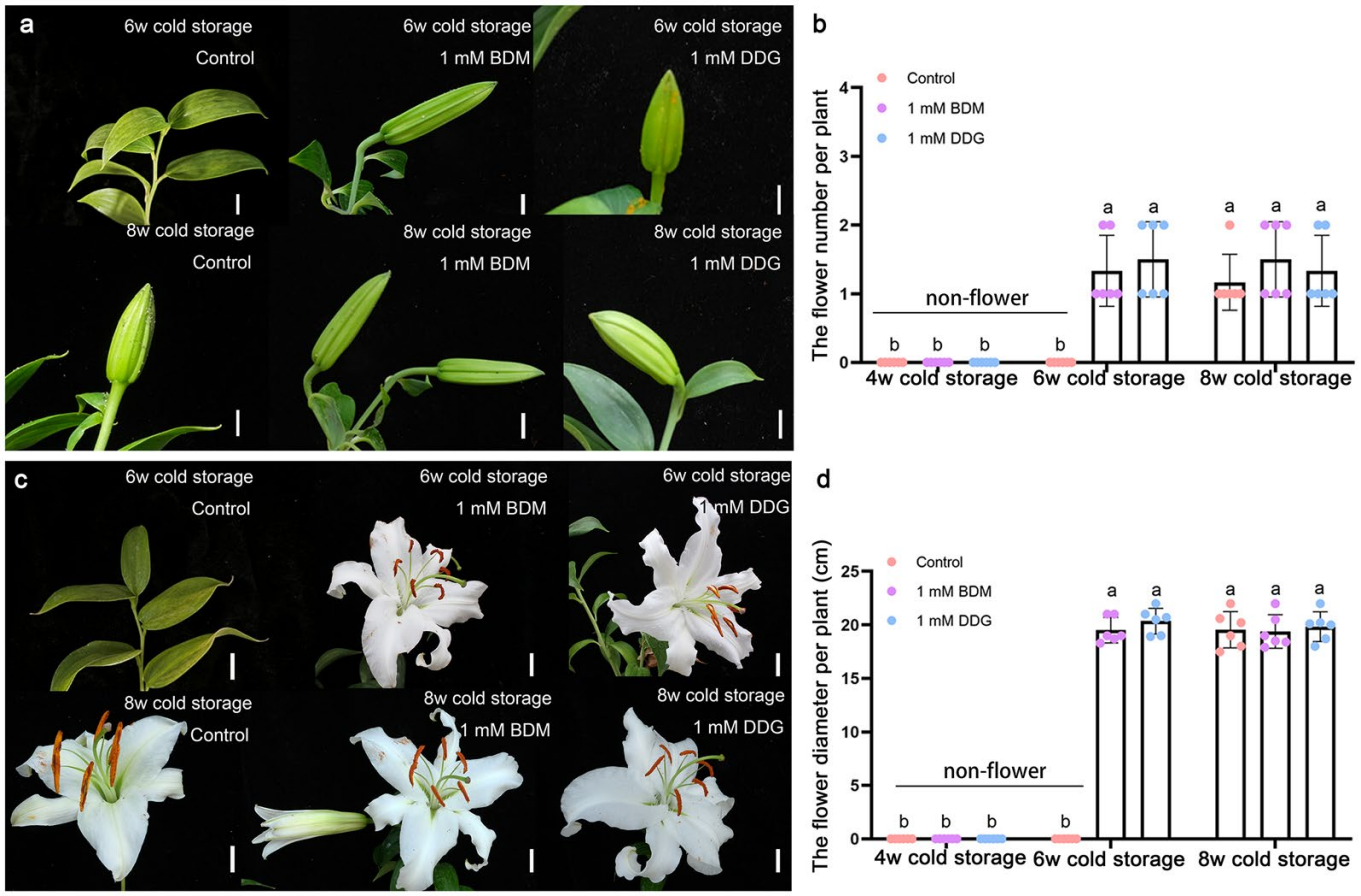
Effects of BDM and DDG on flowering of dormant Siberia bulbs. **a** BDM and DDG accelerated flower development in the absence of sufficient cold storage. Insufficient cold storage (6 weeks of cold storage) resulted in flower abortion while 8 weeks of cold storage allowed develop flowers in the control. The images were taken 24/22 weeks after planting. Scale bars, 1 cm; **b** The number of flowers developed from BDM and DDG-treated Siberia bulbs after storage under the low temperature (4 °C) for 4 weeks, 6 weeks, and 8 weeks. The calculation was performed after 24 weeks of planting. **c**, **d** BDM and DDG did not affect flower size. The upper and lower images were taken when flowers were blooming, 26/24 weeks after planting. Scale bars, 2 cm. The calculation was performed at the full blooming stage. Data in **b** and **d** represent mean ± s.d. of six biological replicates (n = 6 bulbs per sample). Different letters indicate significant differences (*p* < 0.05) by ANOVA Turkey’s HSD tests for pairwise comparisons

As flowering is an essential commercial trait in the lily industry, we finally analyzed the differences in floral organ formation and flower size of the bulbs treated with BDM or DDG (NEM-treated bulbs could not sprout normally (Fig. 6). The results showed that: (1) when bulbs had undergone half of the cold storage (4 weeks), all treated bulbs failed to develop into flowering plants (Additional file 1: Fig. S6); (2) when bulbs were stored for 6 weeks, the control bulbs were unable to develop flowers. In contrast, BDM/DDG-treated bulbs were able to develop flowers and eventually flowered 26 weeks after planting (Fig. 8); (3) when the bulbs had been stored for 8 weeks, the control and BDM/DDG-treated bulbs were able to flower, showing similar flower diameters and numbers. These results suggest that BDM and DDG promote flower transition in bulbs with insufficient cold storage time by accelerating dormancy release, and their effect is comparable to providing an additional 2 weeks of cold storage.

## Discussion

Plasmodesmata is a unique channel that connects plant cells through the cell wall, serving as a crucial pathway for intercellular material transport and information transmission. It facilitates the movement of RNA, metabolites, proteins, and hormones in the form of symplastic transport, playing a vital role in plant development and responses to the environment. PD acts as an essential switch for the maintenance and release of plant dormancy [29]. Notably, soluble sugars, upregulated by low temperatures, can be transported via PD, contributing to the release of dormancy in geophytes, like *Gladiolus hybridus* [27, 30]. In the SAM of poplar, the dynamic transformation (degradation and deposition) of callose controls the opening and closing of PD channels, thereby regulating the release and maintenance of dormancy [6]. External factors, such as temperature, light, and biotic stress, as well as endogenous hormones and reactive oxygen species, are involved in regulating the dynamic transformation of callose. In birch and poplar, exposure to short days in autumn induces changes in SAM cells with an increase in callose content, resulting in blocked PD channels. However, during the winter, the callose content decreases, leading to the dredging of PD [31]. Low-temperature exposure triggers bulb growth transition in lily and this process is accompanied by the opening of PD, fast intercellular communication, and increased substance transport [7]. Furthermore, low-temperature vernalization also affects PD activity. During this period, *FT* mRNA and protein can be transported through phloem or PD channels. Therefore, the dredging of PD promotes the transport of *FT* to the SAM, ultimately facilitating flowering after low-temperature exposure [7, 32]. In this study, we find that the symplastic transport is promoted by BDM and DDG, while inhibited by NEM (Figs. 2 and 4), and the dynamics of callose deposition play crucial roles in bulb dormancy release, plant growth, and flowering.

Temperature signaling serves as an important environmental cue for plant growth and long-term cold exposure (LTCE) is especially crucial for dormancy release and vernalization [33, 34]. Insufficient cold exposure time caused insufficient dormancy release, low seed/bud germination, and failures of flower transition [34–36]. In woody plants, LTCE mediates dormancy release and flower differentiation by repressing the positive feedback between *SVL (SHORT VEGETATIVEPHASE-Like)* and *NCED (9-cis-epoxycarotenoid dioxygenase)*, resulting in reduced endogenous ABA content [4, 37, 38]. Moreover, LTCE induces callose degradation and PD opening by accelerating *FT mRNA* in the SAM, leading to dormancy release and floral initiation in *Populus* and Gentian [31, 39]. Additionally, LTCE significantly affects the levels of various endogenous substances, including hormones and carbohydrates. LTCE downregulates ABA content and upregulates GAs and cytokinins, which play a role in regulating PD transport during dormancy release [31, 34, 40–42]. Soluble sugars are transported by both PD and sugar transporters to the SAM, where they serve as energy resources for cell division and differentiation, promoting dormancy release and plant development [27, 43]. Here, we demonstrated BDM and DDG can partially replace low-temperature storage to promote bulb dormancy release, sprouting, plant growth, and flowering in lilies. This effect is attributed to the accelerated intercellular communication and soluble sugars in SAMs (Figs. 3, 4, 5, 6 and 7). Sucrose positively regulates bulb dormancy release and can partially alleviate the inhibition effect of NEM on sprouting **(**Fig. 3**)**. The effectiveness of 1mM BDM/DDG is similar to providing an additional 2 weeks of cold storage. BDM and DDG not only promote plant growth in bulbs with 6 weeks of cold storage but also promote flower development for these bulbs (Figs. 7 and 8). This finding suggests that BDM and DDG may play a positive role in dormancy release and vernation by promoting *FT* mRNA in SAMs and flower transition afterward (Fig. 8, Additional file 1: Fig. S4). Nevertheless, 1 mM BDM/DDG fails to fully promote the growth and flower transition in bulbs with only 4 weeks of cold storage (Figs. 7 and 8; Additional file 1: Fig. S6), suggesting the PD channel is not completely unblocked in these cases. A higher concentration of BDM/DDG may be needed to recover the growth and flower transition in bulbs with 4 weeks of cold storage. NEM is an efficient inhibitor for bulbs, bulbils, and bulblets that can be used for delayed cultivation (Figs. 1 and 5). However, NEM-treated samples show the undesired rosette-leaf phenotype in the production of cut flowers (Fig. 7). Further investigation is needed to adjust the usage of NEM in such scenarios.

## Conclusion

In summary, this study reveals the significant roles of callose-related regulators, BDM, DDG, and NEM, in regulating dormancy in lily bulbs. BDM and DDG facilitate the degradation of callose between cells, leading to the opening of PD and increased symplastic transport (such as soluble sugars and *FT* mRNA) in SAMs, thereby promoting bulb dormancy release. Additionally, BDM and DDG can compensate for the phenotypes of growth arrest, stunted growth, and failed flower development in dormant bulbs due to insufficient low-temperature storage time. Conversely, NEM exhibits functions opposite to those of BDM and DDG. BDM, DDG, and NEM treatments with the vacuum pump can be potentially used in the off-season production of lily flowers.

## Materials and methods

### Plant materials

Axenic and dormant Siberia lily bulblets were cultivated on Murashige and Skoog (MS) medium before treatment. Dormant bulbils of *Lilium*. *lancifolium* were harvested after plants were withered. Deep dormant Siberia bulbs were harvested in May at Nanping city (118.17 N,501 26.65E), Fujian province.

### NEM, BDM, and DDG treatment and sucrose treatment

**Fig. 9:**
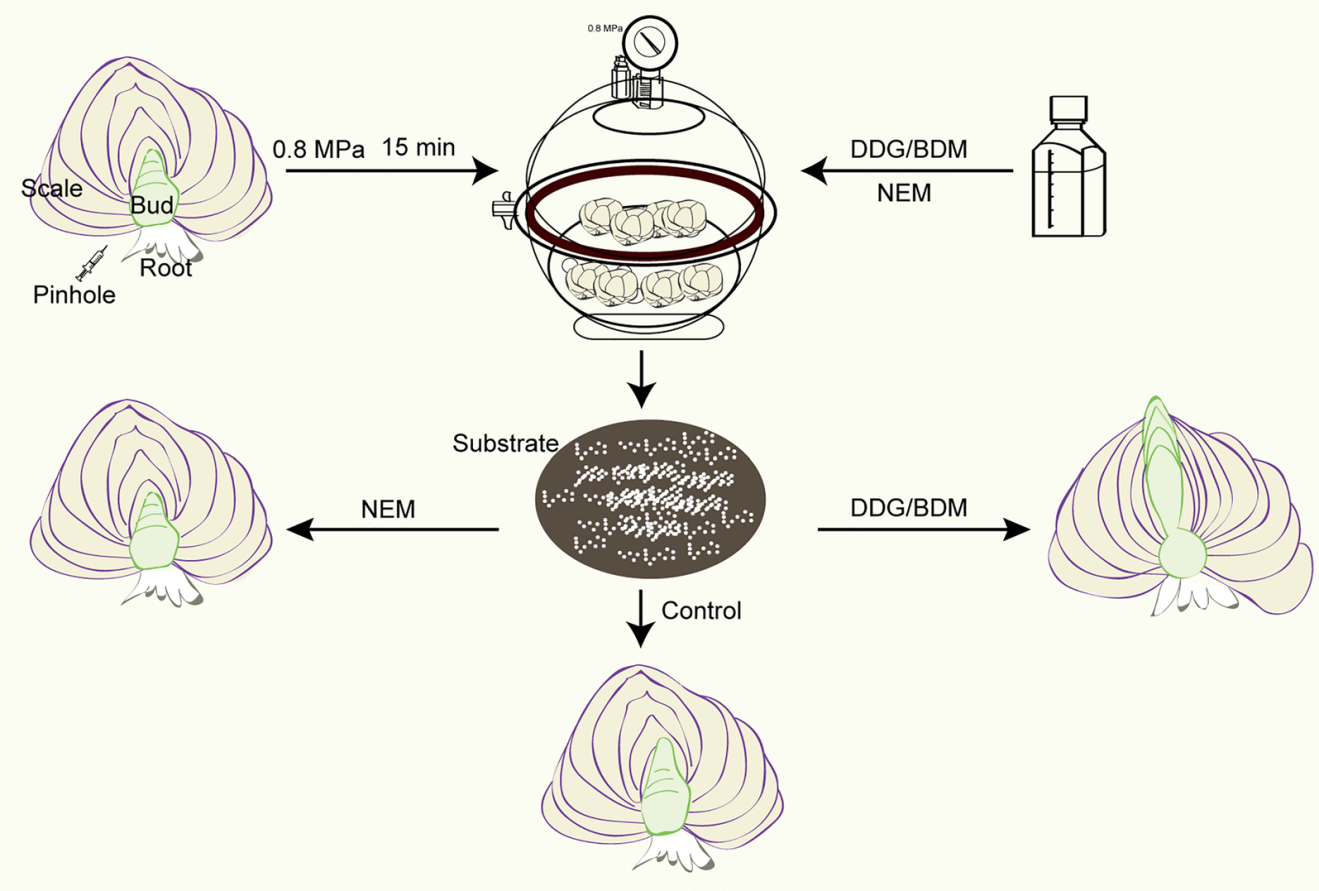
A schematic diagram of the processing method of treating bulbs by NEM, BDM, and DDG. First, use a syringe needle to make punctures near the bud area of the dormant lily bulb. Next, immerse bulbs in a solution containing NEM, BDM, or DDG, and apply a vacuum pressure of 0.8 mPa for 15 min using a vacuum pump. Finally, plant the treated bulb into the soil substrate

A schematic diagram of the process of treating bulbs (Fig. 9). The Siberia bulbs were punctured 10–15 holes at the base plate region by using syringe needles and then immersed in 1mM NEM, BDM, or DDG solution for vacuuming. Pumping the air pressure to 0.8 MPa and maintain the state for 15 min before slowly deflating the air for 15 min to return to the standard atmospheric pressure. ddH_2_O was used as the control. All treated bulbs were embedded in the moist soil substrate and stored at 4 °C. Bulbs were observed and planted after 4 weeks, 6 weeks, and 8 weeks of cold storage. The experiment was performed in at least three biological replicates.

The Siberia bulblets with consistent growth status were cultivated on MS medium containing 1 mM NEM, 1 mM BDM, or 1 mM DDG. MS medium was used as the control. *L*. *lancifolium* bulbils with consistent growth status were treated with 1 mM NEM, 1 mM BDM, or 1 mM DDG by vacuum pump as described above and ddH_2_O was used as the control. All treated bulbils were embedded in the moist soil substrate and stored at 4 °C. The experiments were performed in three biological replicates.

To determine the effect of sugar in combination with NEM, BDM, and DDG on bulblets sprouting, dormant Siberia bulblets were placed on MS medium containing 1mM NEM, 1mM BDM, or 1mM DDG in combination with 30 g/L sucrose or 50 g/L sucrose for a duration of 5 weeks under 16/8 h light/darkness. The experiments were performed in nine independent bulblets for each treatment.

### Phenotype observation and statistics

The germination of Siberia bulblets was observed after 6 weeks of treatment, and the germination refers to bulblets/ bulbils with leaves over 1 cm in length. The growth of *L*. *lancifolium* bulbils was observed 20 weeks after treatment, the bud length is the length from the top of the bud to the budding site of the bulbil. The dormancy release of Siberia bulb was indicated by the bud length/bulb length, the higher ratio represents the faster bulb dormancy released.

### PD transmission electron microscopy (TEM) imaging

SAMs of NEM, BDM, and DDG-treated Siberia bulblet were fixed with 2.5% (w/v) glutaraldehyde overnight at 4 °C. The samples were washed with 0.1 M phosphate buffer (pH 7.0), and post-fixed with 1% (w/v) osmium tetroxide. The permeation-treated samples were embedded with Spurr’s embedding agent and heated at 70 °C overnight. Ultrathin sections of 70 nm thickness were obtained using a LEICA EM UC7 ultramicrotome and were transferred onto 200 mesh formvar/carbon-coated nickel grids (Gilder). The samples were stained with saturated uranyl acetate and 0.2% (w/v) lead citrate at room temperature. The sections were visualized by a Hitachi H-7650 electron microscope. Similar results were observed in three independent samples.

### Callose deposition at PD by using aniline blue staining

SAMs of NEM, BDM, and DDG-treated Siberia bulblet were used for the aniline blue staining and the experiment was performed as previously described [44]. In brief, SAMs were fixed in formalin solution, dehydrated in gradient ethanol, and finally embedded in paraffin wax. The fixed SAM was stained with aniline blue. The image was observed and pictured by ZESSI 710 under the DAPI field. The content of callose was measured by ImageJ. Three biological replicates were performed.

### RNA extraction and RT-qPCR

The SAM of lily bulblets treated with NEM, BDM, or DDG were used for total RNA extraction. Total RNA was extracted using RNA-easy Isolation Reagent (Vazyme). 1 ng total RNA was used to synthesize the first-stand cDNA by HiScript III Kit (+ gDNA wiper; Vazyme). For RT-qPCR, the process was performed with Step One Plus real-time PCR system (Applied Biosystems) using ChamQ Universal SYBR qPCR Master Mix (Vazyme). Expression of *LoCALS3* was used for normalization. *LoFP (F-BOX FAMILY PROTEIN)* served as the internal reference gene. The following thermal profile was used for all RT-qPCRs: 95 °C for 15 min, followed by 40 cycles of 95 °C for 15 s, 60 °C for 30 s, and 72 °C for 30 s. The melt curve analysis was performed by gradually increasing the temperature from 60 to 95 °C at 0.05 °C /s. *LoFP* Forward primer and reverse primer were TCGCCTACATCGCTAACC and TTCCCAATAATCGCAAGACC, respectively. *LoCALS3* forward primer and reverse primer were AGGAAGCAGGCTTACACAGT and TGGCATCCAAGACCATTTGC, respectively. *LoFT1* forward primer and reverse primer were CGCCGAGTCCAAGCAATCCA and TTAGGCCGTGGGCTCTCGTA, respectively.

### Carboxyfluorescein diacetate labeling

To determine the effect of NEM, BDM, or DDG on symplastic transport in SAMs of bulbs, symplastic fluorescent dye carboxyfluorescein (CFDA) was used to observe sugar symplastic transport as previously described with modification [7]. A 50 mg/L work solution of CFDA (APExBIO, #C4995, Houston, USA) was prepared in DMSO. 50 µL work solution was directly loaded into the wedge-shaped block on the abaxial surface of the outer bulb scales and soon fixed with polythene film and aluminum foil. The treated bulblets were incubated for 3 h to allow for CFDA transport. The SAMs were then taken from the bulblets and sliced into 60 μm thin sections by a frozen slicer (Leica CM1850). Subsquently, a fluorescence microscope (Zeiss LSM710) was used to monitor the movement of the CFDA fluorescence under the GFP field. The signals of CFDA were measured by ImageJ. Three biological replicates were performed.

### Fluorescent esculin substrate labeling

To determine the effect of NEM, BDM, or DDG on apoplastic transport in bulbs, apoplastic fluorescent dye esculin were used to stimulate sugar apoplastic transport as previously described with modification [45]. Esculin (MedChemExpress, CAS No.: 531-75-9) was prepared as a 50 mg/L work solution in DMSO. The plates of NEM/BDM/DG-treated bulblets were submerged in the esculin solution for 12 h to allow for esculin transport. The SAMs were taken from the bulblets and were slice into 60 μm thin sections by a frozen slicer (Leica CM1850), after which a fluorescence microscope (Zeiss LSM880) was used to monitor the movement of the esculin fluorescence under DAPI field. The intensity of esculin (Blue fluorescence intensity) was measured by ImageJ. The experiment was performed in at least three biological replicates.

### Soluble sugar content detection

The soluble sugar of SAM was detected by using a plant-soluble-sugar detecting kit (Solarbio). In brief, 200 mg of sample was extracted with 1 mL distilled water at 90 °C for 10 min. After cooling, the resulting supernatant was collected by centrifugation (8000 × *g*, 10 min) and diluted to 10 mL with distilled water. Detection and calculation of soluble sugar content was assayed as described in the instruction. The experiment was conducted with three biological replicates (n = 3 SAMs per sample).

### Statistical analysis

Statistical analyses were performed using one-way analysis of variance (ANOVA) followed by Tukey’s HSD tests for pairwise comparisons. GraphPad Prism (version 8.0.2) and DPS Statistics version 9.01 were used for analysis.

### Supplementary Information

**Additional file 1: Figure S1. ** Dormant bulblets and bulbils used for the sprouting test. **Figure S2.** Effects of NEM, BDM, and DDG on apoplastic transport in SAM of Siberia bulblets. **Figure S3.** Standard curve of soluble sugar contents. **Figure S4.** The expression of LoFT1 in bulblets’ SAMs 6 weeks after treatment with NEM, BDM, and DDG. **Figure S5.** Dormant Siberia bulbs used for the treatments. **Figure S6.** The effect of BDM and DDG on flower transition in bulbs with 4 weeks of cold storage.

## Data Availability

The authors confirm that the data supporting the findings of this study are available within the article and its supplementary materials.
