# Supplementary material for: Regulating bulb dormancy release and flowering in lily through chemical modulation of intercellular communication
Source: Plant Methods. 2023 Nov 28;19:136. doi: 10.1186/s13007-023-01113-y (PMC10683273; doi:10.1186/s13007-023-01113-y)
Supplement: Supplementary file 1 — Additional file 1: Figure S1. Dormant bulblets and bulbils used for the sprouting test. Figure S2. Effects of NEM, BDM, and DDG on apoplastic transport in SAM of Siberia bulblets. Figure S3. Standard curve of soluble sugar contents. Figure S4. The expression of LoFT1 in bulblets’ SAMs 6 weeks after treatment with NEM, BDM, and DDG. Figure S5. Dormant Siberia bulbs used for the treatments. Figure S6. The effect of BDM and DDG on flower transition in bulbs with 4 weeks of cold storage. [file 13007_2023_1113_MOESM1_ESM.pdf]

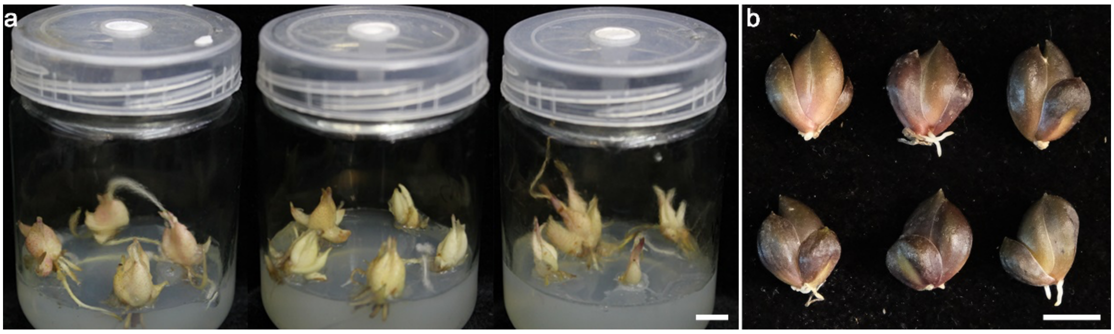

Fig. S1 Dormant bulblets and bulbils used for the sprouting test. Dormant sterilized Siberia bulblets (**a**) and weak dormant bulbils of *Lilium Lancifolium* (**b**) were used for the sprouting test with NEM, BDM, or DDG. Scale bars, 1 cm.

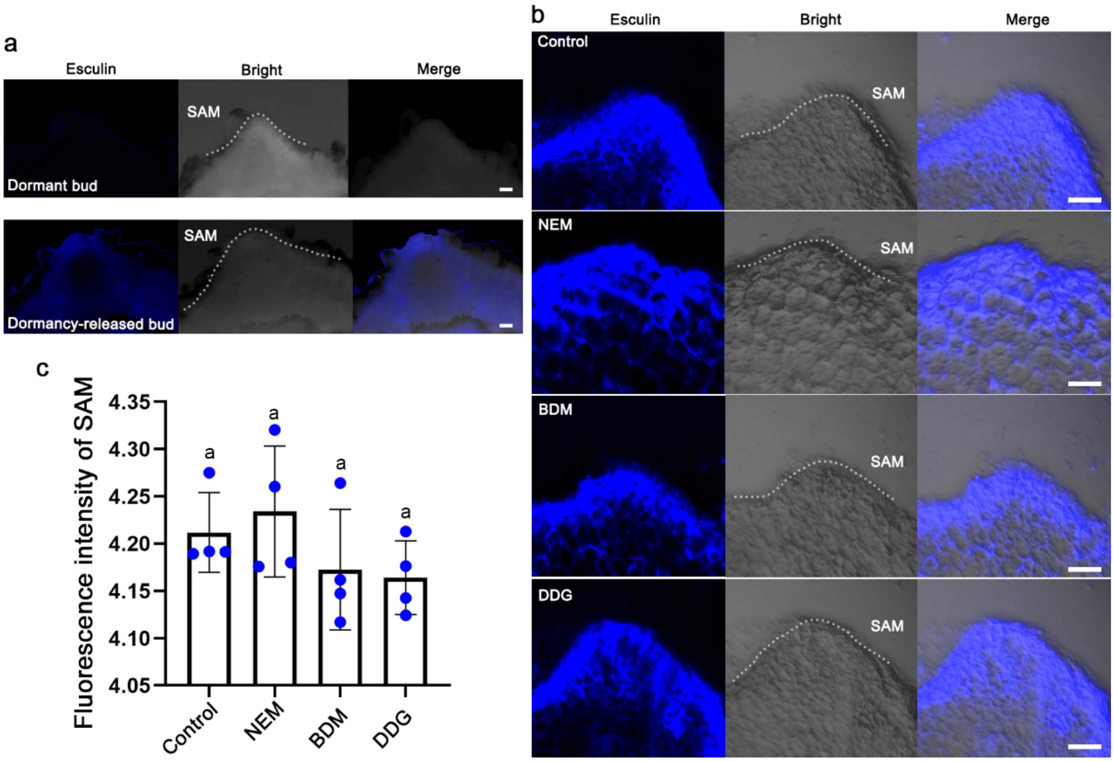

Fig. S2 Effects of NEM, BDM, and DDG on apoplastic transport in SAM of Siberia bulblets. **a** Simulation of apoplastic transport by esculin in central buds of dormant and dormancy released bulbs. Compared with dormant bud, the apoplastic transport capacity is enhanced in dormancy released bud. Scale bars, 200 μm**,** SAM: shoot apical meristem. **b,c** Simulation of apoplastic transport by esculin in central buds of NEM, BDM, and DDG-treated bulblets **(b)**. No significant difference was observed among the various treatments **(c)**. Scale bars, 100 μm, SAM: shoot apical meristem. The blue fluorescence of esculin and ImageJ software were used for quantification. The data represent mean ± s.d. of 4 biological replicates (n=3 slices per sample). The same letter indicates no significant differences (p > 0.05) by ANOVA Turkey's HSD tests for pairwise comparisons.

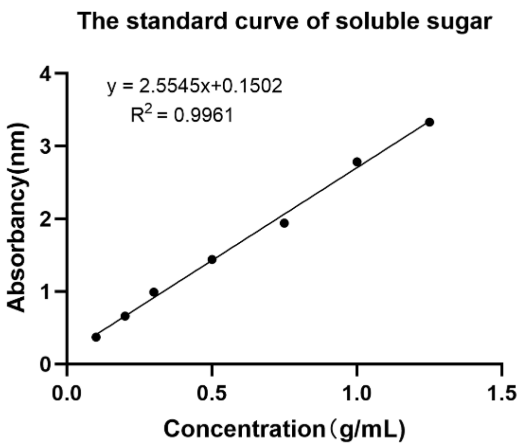

Fig. S3 Standard curve of soluble sugar contents.

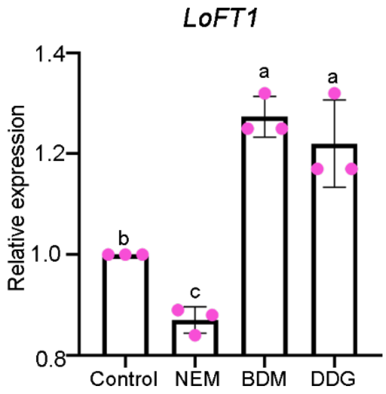

Fig. S4 The expression of *LoFT1* in bulblets' SAMs 6 weeks after treatment with NEM, BDM, and DDG. Data are presented as mean ± s.d. of three biological replicates. Different letters indicate significant differences ($p < 0.05$) by ANOVA Turkey's HSD tests for pairwise comparisons.

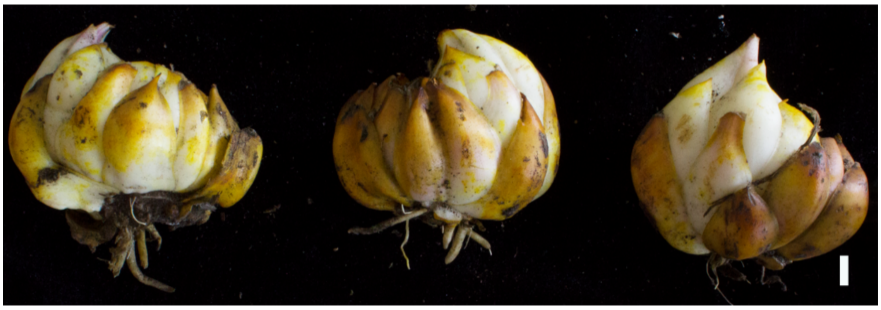

Fig. S5 Dormant Siberia bulbs used for the treatments. Deep dormant Siberia bulbs in Fujian were harvested in May before cold storage was used in this study. Scale bar, 1 cm.

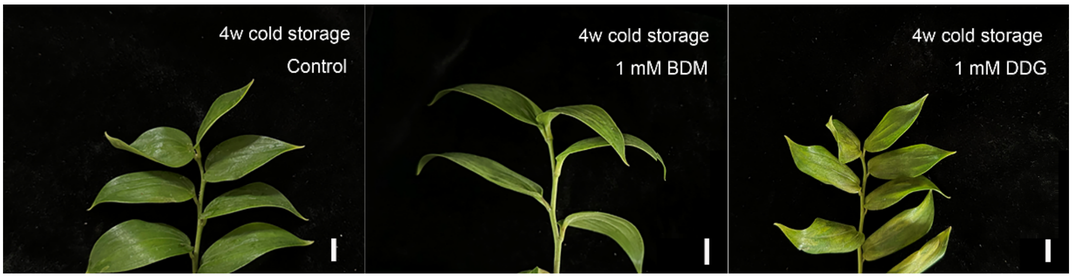

Fig. S6 The effect of BDM and DDG on flower transition in bulbs with 4 weeks of cold storage. Insufficient cold storage (4 weeks of cold storage) resulted in flower abortion in BDM/DDG-treated bulbs. The bulbs were treated with 1mM BDM or DDG before cold storage. The treated bulbs were potted on the soil after 4 weeks of cold storage. The ddH_2O treatment was used as the control. The images were taken 28 weeks after planting. Scale bars, 1 cm.
